# Combining Pulsed and Conventional Radiofrequency Ablation for Resistant Morton’s Neuroma: A Case Report and Literature Review

**DOI:** 10.5812/aapm-161482

**Published:** 2025-05-19

**Authors:** Karim Hemati, Sajede Salehi, Parnian Hemati

**Affiliations:** 1 Pain Research Center, Department of Anesthesiology and Pain Medicine, Iran University of Medical Sciences, Tehran, Iran; 2 Department of Pain Medicine and Anesthesiology, Baqiyatallah University of Medical Sciencs, Tehran, Iran; 3 Department of Anesthesiology and Pain Medicine, Iran University of Medical Sciences, Tehran, Iran; 4 People's Friendship University of Russia, Rudn University, Moscow, Russia

**Keywords:** Case Report, Conventional Radiofrequency, Fore-foot Pain, Morton’s Neuroma, Neuropathy, Pulsed Radiofrequency

## Abstract

**Introduction:**

Morton's neuroma is a painful, degenerative neuropathy that is initially managed with conservative treatments. In refractory cases, surgical excision is typically indicated. Minimally invasive percutaneous procedures provide a viable alternative to surgery.

**Objective:**

While both pulsed radiofrequency (PRF) and conventional radiofrequency (CRF) have been individually reported as effective treatments for Morton’s neuroma, we aim to utilize a combination of PRF and CRF for pain management.

**Case Presentation:**

We report the case of a 61-year-old male with a five-year history of right foot Morton’s neuroma, presenting with severe pain that was refractory to conservative management, including multiple corticosteroid injections. Radiofrequency was performed as follows: 5 minutes at 42°C, 1 minute at 60°C, and 1 minute at 70°C.

**Result:**

The patient experienced significant pain relief, with follow-up Numerical Rating Scale (NRS) of 1 at two weeks, 1 at one month, and 2 at seven months post-treatment.

**Conclusions:**

This case suggests that the combined application of PRF and CRF may serve as a promising alternative for managing refractory Morton’s neuroma.

## 1. Introduction

Morton’s neuroma is a degenerative neuropathy affecting the medial or lateral branches of the plantar nerve, typically located within the inter-metatarsal spaces, most commonly between the third and fourth, or second and third metatarsals (1). Patients often report forefoot pain, characterized as sharp, burning, and associated with numbness on either the dorsal or plantar aspects of the forefoot (2). In some cases, the condition may be asymptomatic, with symptoms sometimes initiating after forefoot trauma. Pain is exacerbated by activities such as walking, running, or wearing high-heeled shoes.

Initial management is conservative, including the use of metatarsal pads, orthotic devices, and analgesics. If conservative treatment proves ineffective, surgical excision may be considered. Minimally invasive alternatives, such as percutaneous injections of corticosteroids, local anesthetics, botulinum toxin, alcohol for chemical neurolysis, radiofrequency ablation, or cryo-ablation, offer potential treatments in place of surgical removal (3, 4).

## 2. Objectives

Although both pulsed and conventional radiofrequency have shown efficacy in treating Morton’s neuroma (5, 6), we chose to combine these two modalities in the management of our patient's condition.

## 3. Case Presentation

The case involves a 61-year-old male with a 5-year history of burning pain in the plantar surface of the right forefoot, which worsened with prolonged walking or standing. He was diagnosed with Morton’s neuroma in the second interdigital space following MRI and electrodiagnostic studies. The MRI ruled out joint instability, occult masses, or tumors, while nerve conduction studies revealed abnormal amplitude and peak latency values in the interdigital nerve. The pain was unresponsive to medical therapy, and the patient underwent approximately five percutaneous corticosteroid injections combined with local anesthetics, which provided insufficient and short-lived relief.

He was referred to our pain clinic due to severe pain that prevented him from walking even short distances, with a Numerical Rating Scale (NRS) score of 10 (on a scale of 0 = no pain, to 10 = the most severe pain experienced). The pain was localized to the anterior half of the plantar surface of the forefoot and was exacerbated by walking and physical touch. Physical examination reproduced symptoms upon performing Mulder's maneuver, which elicited pain when compressing the metatarsal heads while simultaneously applying pressure to the interdigital space. This condition significantly impacted his social life, despite daily use of diclofenac twice per day and pregabalin 75 mg twice per day.

We opted to manage the patient's Morton’s neuroma using a combination of pulsed and conventional radiofrequency ablation. The patient was referred to our pain operating room on 2024/08/25.

### 3.1. Procedure

He was positioned in the supine position with a pillow placed under the right knee to slightly flex the leg. Standard monitoring (electrocardiography, noninvasive blood pressure, and pulse oximetry) was applied. The affected foot was prepared and draped in a sterile manner, and the interdigital space of the affected area was marked with a sterile marker. A high-frequency (5 - 13 MHz) linear ultrasound transducer (Sonosite S-Nerve, USA) was placed transversely to identify the second and third metatarsal heads. The neuroma was localized under ultrasound guidance.

A 1 cc injection of 1% lidocaine was administered at the entry point on the dorsal surface of the foot for local anesthesia. A 20-gauge radiofrequency cannula (COSMAN cannula CCTM, 10 cm 20G Netherlands) with a 5-mm active tip was then introduced through an out-of-plane approach into the inter-metatarsal space adjacent to the neuroma, under ultrasound guidance. After confirming the correct needle tip placement, the radiofrequency probe was inserted. Radiofrequency was performed as follows: 5 minutes at 42°C, 1 minute at 60°C, and 1 minute at 70°C, using a radiofrequency generator (COSMAN MEDICAL, INC. RFG-4, made in USA).

After the procedure, the needle was removed, pressure was applied to the site, and the patient was transferred to the recovery room for monitoring.

## 4. Results

At the 2-week and 1-month follow-ups, the patient reported more than 90 percent pain relief, with a NRS score of 1. He was able to touch his foot and walk with slight tolerable pain. At the three-month follow-up, the pain relief was sustained, and the patient had returned to work. The patient needed to consume diclofenac 100 mg daily in the first two weeks, which was successfully ceased in the subsequent follow-ups. This positive trend persisted at the seven-month follow-up, with more than 70% pain reduction (NRS = 2), emphasizing the long-lasting benefit of this approach.

## 5. Discussion

Morton’s neuroma, first described by Thomas George Morton in 1876 (7), is not a true neuroma but rather fibrosis of the interdigital nerves secondary to repetitive irritation or pressure on the nerves. These factors contribute to degenerative changes such as endoneural edema formation, axonal injury, vascular changes, thickening of the nerve, and perineural fibrosis (8). The third interdigital space is more affected due to its thicker diameter, making it more prone to compression and trauma (9). Patients usually complain of plantar pain and tenderness between the affected interdigital space, accompanied by burning and electric sensations, which are aggravated by walking or wearing tight-fitting shoes (10), causing functional disability.

Diagnosis is based on history, clinical examination, and imaging studies to rule out other differential diagnoses (11). Morton’s neuroma is initially managed by reducing pressure on the foot by avoiding tight-fitting or high-heeled shoes and using non-steroidal anti-inflammatory drugs or anticonvulsants. The application of heat or cold compresses may be beneficial. If these conservative treatments fail, injection of local anesthetics and steroids is indicated (12). However, steroid injection may worsen the pressure on the nerve due to atrophy of subcutaneous fat and the plantar fat pad (13).

Among minimally invasive procedures, radiofrequency has gained acceptance in the management of Morton’s neuroma, as demonstrated by Connors et al., who showed the efficacy of radiofrequency (14). In this case, due to the severity of the symptoms, which were resistant to treatment and multiple injections, we opted to use both pulsed and conventional radiofrequency (as neuromodulation and nerve lesion, respectively) simultaneously. Masala et al. demonstrated the safety and efficacy of conventional radiofrequency ablation (90 seconds per cycle at 85°C) in 52 patients, with our case responding similarly to treatment (15). In a systematic review, Llombart-Blanco et al. concluded that higher temperature settings (≥ 85°C) and fewer radiofrequency cycles (≤ 3) resulted in greater improvement in Visual Analog Scale (VAS) scores compared to more than three cycles (16).

While conventional RF can be used at higher temperatures such as 85 - 90°C (15), due to the limited space of the targeted site in this patient and our plan to combine conventional radiofrequency following 5 minutes of pulsed radiofrequency, we decided to use the lower limit of the conventional temperature spectrum. Additionally, Brooks et al. highlighted the superior outcomes of three cycles of radiofrequency ablation compared to two cycles (17). These findings are consistent with our case, which showed a successful response to a combination of three cycles with both higher and lower temperature settings.

### 5.1. Conclusions

In conclusion, combining pulsed and conventional radiofrequency ablation offers a promising and safe alternative approach for improving both pain relief and overall function in resistant Morton’s neuromas. The combination of pulsed and conventional radiofrequency ablation, along with fewer cycles (≤ 3), appears to provide long-lasting pain relief, as evidenced by over 70% pain reduction at the seven-month follow-up. This approach offers a less invasive alternative to more aggressive surgical options. Future studies, including larger clinical trials, are necessary to further validate the combination of pulsed and conventional radiofrequency for resistant Morton’s neuroma and establish optimal treatment protocols.

## Data Availability

The dataset presented in the study is available on request from the corresponding author during submission or after publication.
